# Research Advances on Cadmium-Induced Toxicity in Hepatic Macrophages

**DOI:** 10.3390/toxics14010057

**Published:** 2026-01-07

**Authors:** Jiongfei Chen, Zhaoan Wang, Wangying Li, Shibo Ying

**Affiliations:** 1 Department of the Fourth School of Clinical Medicine, Zhejiang Chinese Medical University, Hangzhou 310053, China; 2 School of Public Health, Hangzhou Medical College, Hangzhou 310013, China

**Keywords:** heavy metal, cadmium, hepatic macrophage, hepatotoxicity, Kupffer cells, immunometabolism

## Abstract

Cadmium (Cd) is a highly toxic and pervasive environmental pollutant that exerts detrimental effects on human health through diverse biochemical and molecular mechanisms. As a vital metabolic organ, the liver harbors macrophages that play a crucial role in maintaining hepatic health and function. Current research has paid relatively little attention to the role of macrophages in liver injury induced by heavy metal exposure. This review summarizes current research on the molecular mechanisms underlying cadmium-induced toxicity in hepatic macrophages, focusing on oxidative stress, signaling pathways, gene transcription, and apoptosis. It further examines how cadmium-induced macrophage dysfunction impacts hepatic immunometabolism. Specifically, we detail how cadmium triggers oxidative stress and disrupts intracellular calcium homeostasis, leading to the activation of transcription factors such as *NF-κB* and *Nrf2*, and the subsequent engagement of related signaling cascades. These perturbations alter macrophage polarization (M1/M2), promote cellular damage and apoptosis, and ultimately exacerbate hepatic inflammation and fibrosis. By synthesizing recent advances in this field, this review aims to provide a theoretical foundation and future directions for research, with the goal of informing novel strategies for the prevention and treatment of heavy metal-associated liver diseases.

## 1. Introduction

Cadmium (Cd) is a highly toxic heavy metal notable for its significant bioaccumulation potential. Owing to its long half-life (15–30 years) and high chemical stability, it is resistant to degradation and persists in the environment [1]. Human exposure to cadmium occurs primarily via the gastrointestinal tract, respiratory tract, and skin. Anthropogenic activities, primarily mining, industrial production in sectors such as metal smelting, battery manufacturing, and the production of plastics and pigments, and the application of cadmium-containing fertilizers and sewage sludge in agriculture constitute major sources of soil and water contamination. Subsequently, cadmium enters the food chain through uptake by crops, leading to human exposure [2,3]. Moreover, tobacco smoke constitutes a significant source of cadmium exposure for smokers. The pulmonary absorption efficiency of cadmium is considerably higher than that of the gastrointestinal tract. Consequently, smokers can exhibit blood cadmium concentrations up to five times greater than those of non-smokers [4]. Epidemiological investigations indicate that individuals occupationally exposed to cadmium exhibit significantly higher cadmium concentrations in blood and urine compared to non-occupationally exposed populations, with levels reaching up to 34 µg/L in blood and 62 µg/L in urine. Furthermore, even for residents in areas not considered cadmium-polluted, cadmium intake can exceed the established safety threshold, in some cases by approximately threefold [5].

Following absorption, cadmium rapidly enters the systemic circulation [3], and distributes to various organs, accumulating primarily as metallothionein-bound complexes (CdMT) or inorganic salts. The liver and kidneys alone account for about 50% of the total body cadmium burden [6], with other sites of deposition including the bones, testes, spleen, heart, and lungs. Substantial research connects cadmium exposure to a spectrum of liver pathologies, such as hepatitis, non-alcoholic fatty liver disease (NAFLD), liver fibrosis, and liver cancer [7,8,9,10]. Epidemiological studies indicate that higher blood and urinary cadmium levels are associated with elevated liver enzymes (ALT, AST, GGT), and a greater than three-fold increased mortality risk from liver disease is observed in those with urinary cadmium in the highest quartile [11,12,13]. Furthermore, animal experiments confirm the hepatotoxicity of cadmium, characterized by histopathological alterations including steatosis, zonal necrosis, mixed inflammatory cell infiltration, macrophage hypertrophy, and mitochondrial cristae damage [14,15,16,17]. Previous studies have proposed that cadmium exposure induces hepatotoxicity through two distinct pathways: first, via the direct toxic effects of the heavy metal on hepatocytes combined with ischemia resulting from endothelial cell injury; and second, through the activation of macrophages and the infiltration of inflammatory cells, which triggers a complex inflammatory cascade [18,19].

**Figure 1:**
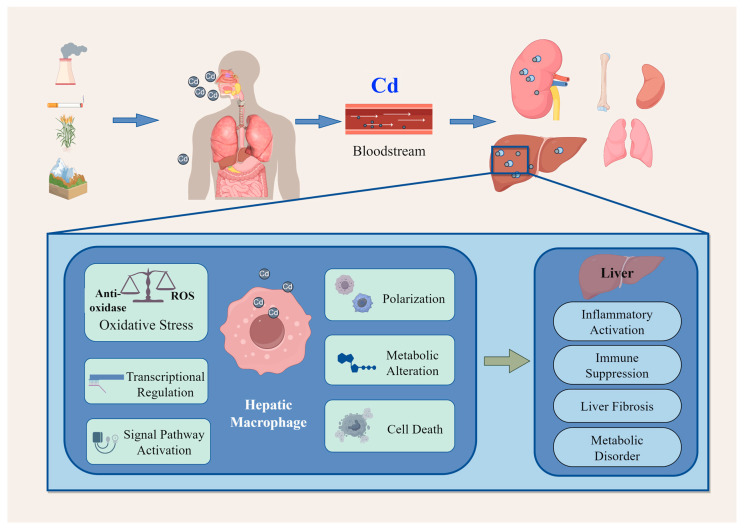
Schematic illustration of cadmium exposure pathways, its adverse effects on hepatic macrophages and subsequent liver injury. This figure is drawn by using the Figdraw platform. The blue arrow corresponds to the intake or delivery of cadmium, and the green arrow represents the resulting hepatic effects of cellular phenotype alterations.

Serving as the body’s first line of defense, macrophages are capable of detecting tissue injury, pathogens, and antibodies. Their pivotal roles in infection clearance, tissue repair, and various inflammatory processes are mediated through phagocytic activity and sentinel functions [20]. Hepatic macrophages primarily consist of resident Kupffer cells (KCs) and monocyte-derived macrophages (MoMФs) recruited from the circulation [21]; they play a crucial role in hepatic immune homeostasis, inflammatory responses, and metabolic regulation, as well as injury repair and fibrosis. Current research focuses on the dual role of macrophages in the pathogenesis of liver diseases such as fibrosis, hepatocellular carcinoma, and chronic hepatitis. For instance, following hepatic injury or infection, the rapid activation of hepatic macrophages and their subsequent release of pro-inflammatory factors mount an inflammatory response. This response serves a protective role by aiding in pathogen elimination in the acute phase; however, its persistence can become detrimental, ultimately promoting chronic inflammation, fibrosis, and carcinogenesis [22,23]. The multifaceted role of hepatic macrophages in liver immune homeostasis, injury response, and the progression and regression of fibrosis positions them as a promising therapeutic target for the intervention of liver diseases. Cadmium activates KCs either directly via their pattern recognition receptors (PRRs) [24] or indirectly through damage-associated molecular patterns (DAMPs) released from injured hepatocytes [12,24]. Internally, cadmium subsequently provokes oxidative stress, inflammation, and metabolic dysregulation in these macrophages [25], see Figure 1.

The toxic effects of cadmium exposure on hepatic macrophages represent a key mechanism in the pathogenesis of cadmium-related liver diseases. This review summarizes recent advances in understanding cadmium-induced macrophage toxicity, aiming to provide novel strategies for the prevention and treatment of heavy metal-induced liver pathologies.

## 2. Effects of Cadmium on Hepatic Macrophages

### 2.1. Oxidative Stress in Macrophages

Oxidative stress is a primary cause of cadmium-induced cytotoxicity and genotoxicity. In vitro studies have demonstrated that cadmium exposure elevates intracellular ROS levels in hepatic macrophages, including superoxide anions (O_2_•^−^), hydrogen peroxide (H_2_O_2_), hydroxyl radicals (•OH), and nitric oxide (NO) [26].

Upon entering the cell, cadmium induces a decrease in the mitochondrial membrane potential, which impedes electron transfer along the respiratory chain. The leaked electrons react with O_2_, generating ROS such as O_2_•^−^. The resulting excessive ROS depletes antioxidant reserves, including glutathione (GSH) [27,28]. Compounding this effect, the high thiophility of cadmium ions promotes their binding to thiol-rich molecules like GSH, a crucial regulator of cellular reduction–oxidation balance [29,30]. Furthermore, cadmium suppresses the expression of major antioxidant enzymes, such as superoxide dismutase (SOD), glutathione peroxidase (GPx), and catalase (CAT) [31], thereby compromising the cellular antioxidant defense system. Additionally, cadmium displaces essential metals such as iron and copper within the cell via molecular mimicry. This leads to an increase in the cytosolic concentrations of these free ions, which in turn participate in Fenton reactions, resulting in the excessive generation of ROS [32,33]. Under these combined mechanisms, the balance between ROS generation and elimination is disrupted, leading to significant oxidative stress.

In macrophages, excessive ROS cause oxidative damage to key biomolecules and activates signaling pathways, thereby driving a range of outcomes from inflammation and altered polarization to apoptosis [26,34,35]. Experimental evidence shows that reducing ROS levels with scavengers markedly lowers inflammatory factors in cadmium-treated KCs. This finding is corroborated in vivo, where ROS inhibition reduces macrophage activation and ameliorates liver tissue damage [26]. These findings collectively suggest that ROS play a critical initiating factor in the pathway leading from cadmium exposure to macrophage activation and liver injury.

### 2.2. Alterations in Gene Transcription

The dysregulation of ROS in macrophages after cadmium exposure induces an adaptive response via the activation of nuclear transcription. This response aims to reestablish homeostasis, resulting in a new functional equilibrium for the cell [36].

Nuclear Factor kappa-light-chain-enhancer of Activated B Cells (*NF-κB*) is a core transcription factor governing inflammatory and immune responses, and its activity is sensitive to the cellular redox state [37,38]. In hepatic macrophages exposed to cadmium, a significant increase in *NF-κB* expression has been observed [26,28,35]. For example, in an in vitro experiment by Peng et al. [26] utilizing a KC model, cadmium exposure was found to induce substantial ROS generation, oxidative stress, and inflammation. Pretreatment with a ROS scavenger and an *NF-κB* nuclear translocation inhibitor resulted in reduced expression levels of *NF-κB*, caspase-1, and NLRP3.

Furthermore, upregulation of multiple genes involved in inflammation, oxidative stress, and apoptosis has been observed in macrophages following cadmium exposure [12,39]. In a mouse study by Xu et al. [35] cadmium exposure induced inflammatory cell infiltration and polarization of hepatic macrophages in the liver. This was accompanied by a significant upregulation in the mRNA expression of inflammatory cytokines such as TNF-α and MCP-1, as well as pro-/anti-inflammatory cytokines including IL-1α and IL-1β.

Nuclear factor erythroid 2-related factor 2 (*Nrf2*), a key transcription factor in the antioxidant response. It drives the expression of antioxidant factors, thereby protecting cells from oxidative damage [40]. Studies have shown that cadmium-induced oxidative stress leads to reduced *Nrf2* transcription and suppresses the expression of antioxidant enzymes such as HO-1 and NQO-1, further diminishing cellular antioxidant capacity [41,42].For instance, in vitro experiments by Liu et al. demonstrated that cadmium exposure induces rROS) generation in RAW264.7 cells, which in turn suppresses *Nrf2* expression and promotes cellular inflammation [43]. Furthermore, alterations in the *Nrf2* factor following cadmium exposure have also been observed in other cell types, including osteoclasts and renal macrophages [44,45].

In a mouse study utilizing real-time PCR, Horiguchi et al. [46] reported that cadmium exposure induced a slow but sustained accumulation of neutrophils, contrasting with the rapid neutrophil influx triggered by lipopolysaccharide (LPS). Concurrently, cadmium exposure upregulated granulocyte colony-stimulating factor (G-CSF) mRNA expression in hepatic Kupffer cells, a response consistent with the observed pattern of neutrophil infiltration. These findings suggest that cadmium-induced macrophage activation plays a significant role in driving the progression of hepatic inflammation.

Studies in pulmonary macrophages have shown that cadmium exposure upregulates the expression of hypoxia-inducible factor-α (*HIF-α*) while downregulating peroxisome proliferator-activated receptor γ (*PPARγ*). Both are key regulators of macrophage inflammation, with *HIF-α* additionally involved in modulating glycolytic pathways. Transcriptomic analysis has further revealed significant alterations in the expression of metabolism-related genes in macrophages following cadmium exposure, particularly those involved in glycolysis, fatty acid metabolism, and redox reactions. However, experimental data specifically from hepatic macrophages are scarce. Given the critical roles of transcription factors such as *Nrf2* and *HIF-α* in regulating oxidative stress and cellular inflammation, further investigation is warranted to examine their alterations in hepatic macrophages following cadmium exposure.

Regarding epigenetic regulation, research on the alterations induced by cadmium exposure in macrophages remains largely unexplored. Cadmium is known to influence chromatin structure and gene expression by modifying post-translational modifications of histones, such as acetylation, methylation, and phosphorylation [47]. Furthermore, evidence suggests that epigenetic changes in hepatic macrophages can impact their functionality. For instance, a murine study on parasitic infection demonstrated that infection leaves long-term transcriptomic and epigenomic imprints on Kupffer cells, subsequently altering some of their functions [48]. Therefore, future research should prioritize elucidating the dynamic epigenetic features of hepatic macrophages under cadmium exposure and determine how these modifications mechanistically regulate macrophage polarization balance and functional responses.

### 2.3. Dysregulation of Signaling Pathways

Cadmium exposure triggers a rise in cytosolic calcium (Ca^2+^) concentration in macrophages. As a key second messenger, Ca^2+^ activates downstream signaling pathways and participates in the physiological regulation of cellular functions [49]. Cadmium disrupts Ca^2+^ homeostasis by molecular mimicry of calcium transport systems [39]. In macrophages, cadmium exposure activates phosphatidylinositol-specific phospholipase C (PI-PLC), leading to inositol-1,4,5-trisphosphate (IP3) generation. The binding of IP3 to endoplasmic reticulum receptors releases stored Ca^2+^ into the cytosol [50], priming a signaling cascade that drives inflammation and apoptosis.

Cell-based assays demonstrate that cadmium exposure activates multiple signaling pathways in macrophages (See Table 1). The activation of the *NF-κB* pathway plays a critical role in the inflammatory response triggered by cadmium exposure [26,28,35]. In vitro studies on Kupffer cells have demonstrated that cadmium exposure activates the *NF-κB* pathway, which plays a pivotal role in the ensuing inflammatory response. This activation leads to the upregulation of downstream target genes such as IL-1β, IL-6, and TNF-α, and increases NLRP3 expression, thereby promoting the development of inflammation [26].

Phosphatidylinositol 3-Kinase (PI3K)-Protein Kinase B (Akt) signaling transduction also plays a significant role in the activation of macrophages exposed to cadmium [51,52]. In the study on wild boar liver by Cui et al. [53] found that cadmium exposure upregulates *miRNA-21* expression in hepatic macrophages, downregulates *SMAD7* and *TGF*-βexpression, activates the PI3K/AKT signaling pathway, leads to an imbalance in hepatic macrophage polarization, and increases the expression of inflammatory cytokines. In a chicken liver model, Sun et al. discovered that Kupffer cells promote hepatic fibrosis by activating the APJ–AMPK–PGC1α pathway, which in turn stimulates hepatic stellate cells [54].

**Table 1:** Signaling pathways activated in macrophages following cadmium exposure.

Chemical Forms of Cd	Dose	Hepatic Macrophages	Source Species	Signaling Pathways	Toxic Effect	Ref.
CdTe	5–50 nM	KUP5 cells	Mice	*NF-κB* signaling pathway	NLRP3 inflammasome expression; Pro-inflammatory cytokine expression	[26]
CdCl_2_	20 mg/kg	Hepatic macrophages	Pigs	PI3K/AKT pathway	M1 polarization; Pro-inflammatory cytokine expression	[53]
CdTe	1 μM	RAW264.7 cells	Mice	*Nrf2* pathway inhibition	Pro-inflammatory cytokine expression; Mitophagy; Ferroptosis	[42,43]
CdCl_2_	140 mg/kg	KCs	Chicks	APJ-AMPK-PGC1α	activate astrocytes and more hepatic fibrosis	[54]
CdCl_2_	0.1–1 μM	Thioglycollate-elicited macrophages	Mice	MAPK pathway	Promotion of macrophage proliferation	[50]
CdCl_2_	20, 100, 500 μM	J774A.1 murine macrophage cells	Mice	JNK-caspase-3 pathway	Macrophage apoptosis; Growth arrest; Mitochondrial impairment	[55]

Furthermore, the mitogen-activated protein kinase (MAPK) signaling pathway also plays a significant role in cadmium-induced macrophage activation. MAPKs are serine/threonine-specific protein kinases that regulate the activation of various cellular responses to stress, such as proliferation, gene expression, differentiation, mitosis, cell survival, and apoptosis, thereby mediating the cellular stress response [40]. Experimental studies indicate that cadmium-induced ROS and Ca^2+^ overload trigger the phosphorylation of MAPK family members, such as c-Jun N-terminal kinase (JNK), extracellular signal-regulated kinase (ERK), and p38 Mitogen-Activated Protein Kinase (p38). This activation subsequently initiates downstream signaling cascades, thereby modulating critical cellular processes including proliferation, apoptosis, and inflammatory responses [41,42]. However, these experimental data are predominantly derived from studies on murine RAW264.7 cells or mouse macrophages, highlighting a lack of direct investigations into hepatic macrophage.

Cadmium induces the production of reactive oxygen species (ROS) and triggers intracellular calcium overload, two key mediators that subsequently initiate multiple downstream signaling cascades. This process ultimately induces macrophage activation and triggers a range of toxic effects including inflammatory responses and cell death. Future investigations should prioritize the identification of key signaling pathways and their critical regulatory nodes as potential targets for intervention. A deeper exploration of the underlying molecular mechanisms and specific therapeutic targets will establish a theoretical foundation and reveal promising strategies for alleviating cadmium induced toxicity in hepatic macrophages.

### 2.4. Alterations in Metabolic Pathways

Cadmium exposure leads to significant changes in macrophage energy metabolism, especially in glucose and fatty acid metabolism, with direct implications for their polarization and functional state [56]. In an in vitro study by Chen et al. [57] lipopolysaccharide (LPS) stimulation of mouse bone marrow-derived macrophages (BMDMs) was found to upregulate the mRNA and protein expression of multiple M1 polarization-related inflammatory cytokines. This upregulation was significantly suppressed by the application of a glycolysis inhibitor, indicating that glycolytic activity is essential for M1 polarization. Further experiments revealed that enhanced glycolysis in hepatic macrophages activates the STING/TBK1/IRF3 pathway, which plays a critical synergistic role in promoting the pro-inflammatory phenotype of macrophages—a phenotype known to be pivotal in the initiation and progression of liver fibrosis. Lu et al. [58] revealed that through F13A1-PKM2-mediated metabolic reprogramming, hepatic macrophages aggravate MASH-related liver inflammation and injury by both enhancing the Warburg effect to remodel metabolism and facilitating pro-inflammatory factor release via the PKM2/HIF1α axis.

Studies have revealed that cadmium exposure leads to an enhancement glycolysis, characterized by lactate accumulation, elevated levels of glycolytic intermediates, and altered amino acid profiles—specifically, increases in 3-phosphoglycerate, phosphoenolpyruvate, and alanine, alongside reduced synthesis of cysteine and glycine. Concurrently, the expression of key glycolytic enzymes—hexokinase 2 (HK2), phosphofructokinase (PFK), glyceraldehyde-3-phosphate dehydrogenase (GAPDH), pyruvate kinase (PKM), and lactate dehydrogenase A (LDHA)—was markedly upregulated, resulting in significantly elevated lactate levels in the culture medium [59]. In a study by Yao et al. [56] on cadmium-exposed adrenal macrophages in pigs, it was observed that in addition to the upregulation of key glycolytic enzymes, the expression of mitochondrial enzymes involved in the respiratory chain was also significantly elevated—including cytochrome c oxidase subunit 4 (COX4), succinate dehydrogenase complex iron-sulfur subunit B (SDHB), and NADH-ubiquinone oxidoreductase B8 subunit (NDUFB8). These findings indicate a marked enhancement of cellular energy metabolism.

These findings collectively suggest that cadmium exposure may disrupt the energetic state of macrophages, leading to metabolic dysfunction and lactate accumulation, thereby impairing their function and activity. The metabolic reprogramming of macrophages following cadmium exposure leads to intracellular fatty acid dysregulation, which in turn impair cell membrane integrity and function [60]. Olszowski et al. [60] revealed that treating THP-1 macrophages with very low concentrations of cadmium (5–200 nM) led to a significant decrease in the levels of arachidic acid, palmitoleic acid, oleic acid, and linoleic acid, while the level of arachidonic acid was markedly increased. Separately, Dumkova et al. [61] reported that cadmium exposure in mice resulted in Kupffer cells exhibiting an increased amount of smooth endoplasmic reticulum and a reduced amount of rough endoplasmic reticulum, suggesting potential alterations in steroidogenesis or metabolic functions in hepatic Kupffer cells following cadmium exposure.

Current studies revealed that metabolomic alterations in hepatic macrophages influence their phenotype and contribute to liver injury [57,58]. Furthermore, studies on macrophages from other tissues have demonstrated that cadmium exposure induces metabolic changes. However, direct evidence regarding the impact of cadmium on the metabolism of liver-resident macrophages is currently lacking. Therefore, a deeper investigation is needed to elucidate the regulatory mechanisms of metabolism-related genes, changes in metabolic products, and associated functional phenotypes in cadmium-exposed hepatic macrophages. Such studies will provide a theoretical foundation for clarifying the molecular mechanisms underlying cadmium-induced liver injury.

### 2.5. Alterations in Cellular Polarization

Hepatic macrophages possess two classical phenotypes: M1 macrophages are primarily pro-inflammatory, while M2 macrophages are anti-inflammatory and promote tissue repair [62,63]. Phenotypic changes in macrophages influence the pathogenesis of various diseases. In NAFLD and NASH, an increase in M1 macrophages is closely associated with exacerbated liver inflammation and injury, whereas M2 macrophages are linked to hepatic repair and regeneration [64,65]. The polarization of macrophages is orchestrated by a network of signaling pathways, including PI3K/Akt, JAK2/STAT3 and TLR4/*NF-κB*. The activity status of these pathways ultimately dictates the functional and phenotypic characteristics of macrophages [52,66].

Cadmium exposure influences the hepatic immune response by altering the polarization state of macrophages. In a study on porcine liver tissue by Cui et al. [53], cadmium exposure significantly increased the expression of M1 macrophage-specific markers, enhanced M1 activation, and exacerbated inflammatory responses, while polarisation toward the M2 phenotype was suppressed, leading to impaired tissue repair. In contrast, Franzoni et al. reported a decrease in M1 macrophage markers in wild boar MoMΦ with no significant effect on M2 markers following cadmium exposure, suggesting that cadmium inhibits M1 polarisation [67]. Differing from both, Li et al. observed in mouse liver tissue that cadmium exposure increased the expression of markers for both M1 and M2 macrophages, yet overall inflammation imbalance predominated [35]. These discrepancies in research findings may arise from inherent differences across species, including variations in hepatic physiological structure, the composition of macrophage subsets, and cadmium metabolism. Additionally, the distinction between in vivo and in vitro study systems is significant, as in vitro models lack the complex microenvironmental regulation present in the whole organism. Variations in key experimental parameters, such as the dose and duration of cadmium exposure, also likely contribute to the observed differences in macrophage polarization phenotypes. Furthermore, the responses of distinct macrophage subsets within the liver have not been sufficiently differentiated in these studies, making it difficult to identify the primary cellular targets of cadmium exposure. Further research is needed to determine whether alterations in polarisation represent a key mechanism in cadmium-induced liver injury and fibrosis.

### 2.6. Macrophage Death

Cadmium exposure disrupts cellular homeostasis by inducing ROS accumulation and intracellular Ca^2+^ dysregulation, triggering downstream signaling pathways and transcriptional reprogramming as adaptive responses to reestablish functional equilibrium. However, when these perturbations become excessive, they initiate deleterious processes, including endoplasmic reticulum stress, mitochondrial dysfunction, and dysregulated autophagy—that ultimately promote cell death [68].

Furthermore, studies in KCs have shown that cadmium exposure activates the *NF-κB* signaling pathway, induces NLRP3 inflammasome assembly, which cleaves Caspase-1 to induce pyroptosis. This process triggers a robust inflammatory immune response, and pyroptosis is considered a key factor in the initiation and progression of metal-induced liver inflammation [22,61].

In macrophages, cadmium exposure elevates cytosolic Ca^2+^ levels, activating calcium-dependent calpains. These proteases subsequently trigger the caspase family cascade, ultimately inducing apoptosis. Concurrently, calpain activation stimulates the AMPKα-mTOR pathway, initiating macrophage autophagy. Excessive autophagic activity further promotes apoptotic cell death [49,69]. In parallel, studies by Liu et al. demonstrate that cadmium exposure increases ROS levels and suppresses NRF2 expression in macrophages, leading to ERK1/2 phosphorylation and subsequent activation of mitophagy and ferroptosis [42,43].

**Figure 2:**
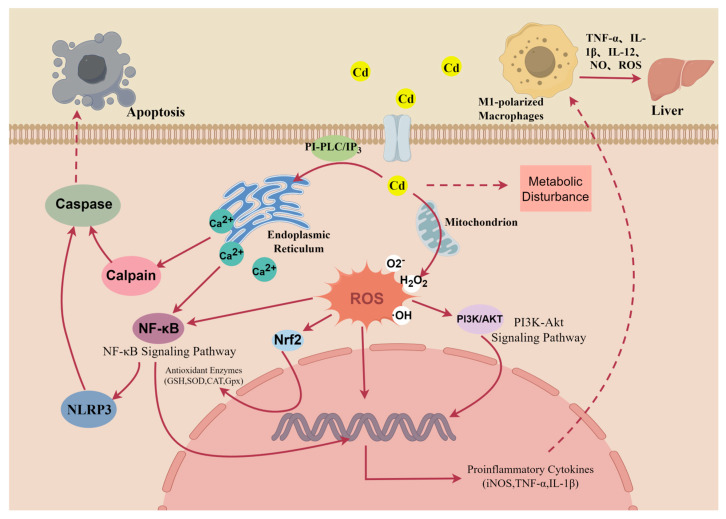
Hypothesis on the molecular mechanisms of cadmium-induced dysregulation in hepatic macrophages. This figure is drawn by using the Figdraw platform. Solid arrows show molecular pathway activation, which triggers the cellular responses depicted by dashed arrows.

While macrophage apoptosis in infectious liver models promotes pathogen clearance and inflammation resolution through ROS/cytokine-mediated recruitment of monocytes and activation of neutrophils [70], its impact in heavy metal-induced sterile inflammation is still unknown. Furthermore, current evidence supporting cadmium-induced macrophage apoptosis is primarily derived from studies on macrophages from other origins. Owing to the functional heterogeneity among different macrophage populations, future studies should focus on defining how cadmium-triggered macrophage death influences liver inflammation, see Figure 2.

## 3. Hepatic Impact of Cadmium-Induced Macrophage Toxicity

As the primary immune cells in the liver, hepatic macrophages are central targets of cadmium toxicity. The toxic effects of cadmium on these cells represent a key mechanism underlying the development of hepatic inflammation, metabolic dysregulation, immune suppression, and fibrosis, all of which contribute to exacerbated liver injury.

Cadmium exposure induces KCs to release abundant pro-inflammatory cytokines, including TNF-α, IL-1β, IL-6, and IL-12, triggering localized inflammatory infiltration in the liver [53,71]. Pro-inflammatory cytokines such as TNF-α activate the death receptor pathway (e.g., Fas/FasL) in hepatocytes, upregulating pro-apoptotic proteins (e.g., Bax) while suppressing anti-apoptotic proteins (e.g., Bcl-2). This imbalance increases the Bax/Bcl-2 ratio, ultimately triggering hepatocyte apoptosis [71].

These cytokines further activate hepatic stellate cells (HSCs), transforming them into myofibroblast-like cells that synthesize excessive extracellular matrix (ECM) components such as collagen (COL) and α-smooth muscle actin (α-SMA) [53,71,72]. Concurrently, M1 macrophages exhibit significantly elevated expression of tissue inhibitor of metalloproteinases1 (TIMP1), while the expression of matrix metalloproteinase2 (MMP2)and matrix metalloproteinase9 (MMP9)—proteases responsible for ECM degradation—is downregulated. This imbalance between enhanced ECM synthesis and reduced degradation leads to excessive collagen deposition in the liver, ultimately promoting hepatic fibrosis [53].

Cadmium exposure compromises the immune function of hepatic macrophages by diminishing their antioxidant capacity and pathogen clearance ability [30]. In vitro studies on peripherally derived MoMΦ indicate that it also downregulates TLR family members and signaling molecules (e.g., MyD88, p65), which diminishes immunorecognition and clearance of pathogens and toxins, thereby elevating the risk of liver inflammation and infection [67].

In the context of hepatic metabolism, metal-induced lipid accumulation is associated with the initiation and progression of liver disease [73,74]. Chronic cadmium exposure inhibits fatty acid desaturation in the liver. In the presence of saturated fatty acids, macrophages exhibit enhanced metabolic activity and elevated oxidative stress under inflammatory conditions, which subsequently promotes hepatic inflammation and fibrosis [75,76].

Hepatic macrophages participate in the metabolism of iron, bilirubin, and other substances in the liver. Chronic cadmium poisoning has been confirmed to cause iron-deficiency anemia [77,78]. Studies have shown that cadmium downregulates the expression of iron transport-related molecules in the duodenum, leading to reduced iron absorption and significantly decreased iron concentrations in the liver [79,80]. As KCs play a crucial role in maintaining iron homeostasis by phagocytosing senescent erythrocytes and recycling free iron [21], further investigation is warranted to determine whether cadmium-induced toxicity to these cells disrupts systemic iron balance.

## 4. Perspectives

There is currently no specifically approved therapy for cadmium poisoning. For liver injury induced by cadmium exposure, studies have shown that certain antioxidants and metabolic modulators can mitigate cadmium toxicity by enhancing the liver’s antioxidant and anti-inflammatory capacities and regulating hepatic metabolism [81,82,83,84]. Given the crucial role of hepatic macrophages in maintaining liver homeostasis and their dual functions in both promoting and suppressing disease progression, they have emerged as promising novel therapeutic targets for liver diseases [21]. Potential strategies include blocking the recruitment of suppressing their activation, monocyte-derived macrophages, and modulating their functional polarization. Such interventions can effectively attenuate macrophage-driven hepatic inflammation, thereby offering novel avenues for treating liver diseases [21,85,86].

Given the pivotal role of hepatic macrophages in the immunometabolic dysregulation induced by cadmium exposure, future research should focus on the following directions to further elucidate the toxicological mechanisms and explore potential countermeasures.

First, elucidate the regulatory mechanisms involving key transcription factors, metabolism-associated genes, and epigenetic modifications in hepatic macrophages under cadmium exposure. Clarify how these regulatory elements influence macrophage functional phenotypes and polarization balance.

Second, investigate the crosstalk between hepatic macrophages and other liver-resident cells (such as hepatocytes, hepatic stellate cells, and sinusoidal endothelial cells) in the context of cadmium exposure. Emphasis should be placed on how paracrine signals (e.g., inflammatory cytokines, chemokines, and metabolic intermediates) secreted by cadmium-activated macrophages mediate intercellular communication and collectively promote liver injury or fibrotic progression.

Third, building on observed macrophage activation in cadmium-exposed animal models, evaluate whether interventions targeting hepatic macrophages (such as modulating polarization balance, inhibiting aberrant signaling pathways, or intervening in metabolic reprogramming) can alleviate cadmium-induced liver injury, and identify the key molecular targets and interventional efficacy involved.

## 5. Conclusions

The impact of heavy metal cadmium exposure on hepatic macrophages represents a complex medical concern. This review collectively demonstrates that cadmium impairs hepatic macrophage function through multiple pathways, triggering a cascade of cellular responses including oxidative stress, inflammatory reactions, metabolic dysregulation, cell death, and functional alterations, thereby compromising overall liver health. These findings provide critical insights into the toxicological mechanisms of cadmium and lay a foundation for future research.

## Data Availability

No new data were created or analyzed in this study. Data sharing is not applicable to this article.
